# Re: Urological surgery in the COVID-19 era: Patient counselling and informed consent

**DOI:** 10.1080/2090598X.2020.1793056

**Published:** 2020-07-13

**Authors:** Elsayed Desouky

**Affiliations:** Department of Urology, Wexham Park NHS Hospital, Berkshire, UK; Department of Urology, Alexandria Main University Hospital, Alexandria, Egypt

Dear Editor,

I read with interest the editorial comment sent by my colleagues regarding the article discussing the process of patient counselling and informed consent in the coronavirus disease 2019 (COVID-19) era [1]. The article was aiming to broadly highlight the topic for all colleague urologists and to raise awareness on how such unprecedented times alter our ‘normal’ practice. These highlights apply to all urological subspecialties, not only during the pandemic surge, but also through the upcoming phase when we are slowly starting to resume our elective surgical activity.

Uro-oncology represents a major proportion of our patients and besides emergency/urgent surgeries, cancer surgeries were the only procedures allowed to proceed, as possible, during this crisis. I do agree with my colleagues regarding the particular point they raised in their comment; the essential role of Uro-oncology multidisciplinary team (MDT) discussion in decision-making amidst the COVID-19 pandemic [1].

As per the Royal College of Surgeons of England, the risk to the surgical patient should be a combined assessment/discussion of the real risk of proceeding under current circumstances vs the real risk of delay. As well, any available alternative treatments must be discussed [2].

At the same time, plans for triage should avoid blanket policies, but rather rely on a day-by-day, data-driven assessment of the changing risk–benefit analysis, taking into account expert clinical opinion. Guidelines provided by national and international urological societies such as the European Association of Urology (EAU), AUA and BAUS, provide helpful guidance during MDT discussion for the best treatment option(s) for the patient, which needs to be tailored based on the available *service provision* in each healthcare facility [3].

Clear documentation of the MDT discussion and outcome is essential, as well as documenting if the MDT treatment plan has been modified in response to the COVID-19 crisis, e.g. skipping BCG maintenance dose [4] or adopting a new protocol of active surveillance of recurrent low-grade muscle-invasive bladder cancer [5]. Similar to informed consent documentation, MDT documentation is of paramount importance from both medical and legal aspects, particularly during this unprecedented time.

At this time of uncertainty, MDT panel discussion of oncology cases provides urologists with the necessary support and provides a solid basis for counselling patients prior to making informed decisions.
